# Temporal regulation of the Mus81-Mms4 endonuclease ensures cell survival under conditions of DNA damage

**DOI:** 10.1093/nar/gkt645

**Published:** 2013-07-30

**Authors:** Irene Saugar, María Victoria Vázquez, María Gallo-Fernández, María Ángeles Ortiz-Bazán, Mónica Segurado, Arturo Calzada, José Antonio Tercero

**Affiliations:** ^1^Centro de Biología Molecular Severo Ochoa (CSIC/UAM), Cantoblanco. 28049-Madrid, Spain and ^2^Centro Nacional de Biotecnología (CSIC), Cantoblanco. 28049-Madrid, Spain

## Abstract

The structure-specific Mus81-Eme1/Mms4 endonuclease contributes importantly to DNA repair and genome integrity maintenance. Here, using budding yeast, we have studied its function and regulation during the cellular response to DNA damage and show that this endonuclease is necessary for successful chromosome replication and cell survival in the presence of DNA lesions that interfere with replication fork progression. On the contrary, Mus81-Mms4 is not required for coping with replicative stress originated by acute treatment with hydroxyurea (HU), which causes fork stalling. Despite its requirement for dealing with DNA lesions that hinder DNA replication, Mus81-Mms4 activation is not induced by DNA damage at replication forks. Full Mus81-Mms4 activity is only acquired when cells finish S-phase and the endonuclease executes its function after the bulk of genome replication is completed. This post-replicative mode of action of Mus81-Mms4 limits its nucleolytic activity during S-phase, thus avoiding the potential cleavage of DNA substrates that could cause genomic instability during DNA replication. At the same time, it constitutes an efficient fail-safe mechanism for processing DNA intermediates that cannot be resolved by other proteins and persist after bulk DNA synthesis, which guarantees the completion of DNA repair and faithful chromosome replication when the DNA is damaged.

## INTRODUCTION

Coping with DNA damage during chromosome replication is a major challenge for cells. An effective DNA damage response is essential for faithful chromosome duplication and genome integrity maintenance, and requires the coordination of checkpoints with multiple pathways and different proteins that are involved in DNA repair (1–5). Among them, structure-specific endonucleases contribute importantly to genomic stability by cleaving some DNA secondary intermediates that are generated during replication-associated repair and need nucleolytic resolution (6–10).

Mus81-Eme1/Mms4 is an evolutionarily conserved structure-specific endonuclease involved in the cellular response to DNA damage (8,11). Yeast cells lacking the catalytic (Mus81) or the non-catalytic subunit (Mms4 in *Saccharomyces cerevisiae*, Eme1 in *Schizosaccharomyces pombe* and human cells) of this complex show sensitivity to a variety of DNA-damaging agents that interfere with DNA replication (12–17). Likewise, mammalian cells deficient in MUS81 or EME1 are sensitive to some agents that cause DNA lesions (18–21), and this endonuclease is necessary for the repair of broken DNA replication forks (RFs) (22,23). Genetic analyses showed that Mus81-Eme1/Mms4 is required for cell viability in the absence of components of the complex formed by the RecQ-helicase Sgs1, Top3 and Rmi1 in budding yeast (BLM-TOPIIIa-RMI1-RMI2 in human cells) (13,15,16,24–28), indicating overlapping functions between Mus81 and RecQ-helicase complexes. Mus81-Mms4 also has functional overlap with the Yen1 resolvase during DNA repair (29–32). The synthetic lethality between Mus81 and RecQ complexes is suppressed by eliminating early steps of homologous recombination (16,24–26), which together with its interaction with Rad54 (14) suggested a role for Mus81-Eme1/Mms4 during recombination-mediated DNA repair (11). Nevertheless, this nuclease is not required for the repair of double-strand breaks through homologous recombination, as *mus81/mms4* mutants are not sensitive to γ-irradiation (12–14). Although it is still unclear which are the substrates of Mus81-Eme1/Mms4 in the cell, a number of biochemical studies have shown that it can cleave different branched DNA structures *in vitro*, all of which are its potential targets *in vivo*. Thus, it is able to act, with different affinity, on model RFs, 3′-flaps (3′-FLs), D-loops, X- and Y-shaped structures (15,16,24,33–41).

The Mus81-Eme1/Mms4 endonuclease has a significant role in the maintenance of genome stability, but its nucleolytic activity needs to be strictly regulated to avoid the undesired opposite effect. The unrestrained cleavage of potential substrates such as RFs and other DNA intermediates would have negative consequences for chromosome replication, and could also lead to the formation of chromosomal rearrangements or high levels of chromatid exchanges. Recent studies in budding yeast have uncovered key features of the regulation of this endonuclease, showing that in an unperturbed mitotic cell cycle its activity is controlled by Cdc28^CDK1^- and Cdc5^PLK1^-dependent phosphorylation of the non-catalytic subunit Mms4 (42,43). This phosphorylation is required for the full nuclease activity of the complex and restricts the function of Mus81-Mms4 to a narrow period in a normal cell cycle, just before chromosome segregation (42,43). A recent report has confirmed this mode of regulation for Mus81-Mms4 and has provided further evidence of its importance for the maintenance of genomic stability, showing that the uncontrolled activation of this endonuclease causes deleterious mitotic crossovers and premature processing of DNA intermediates (44). Human Mus81-Eme1 is also regulated through phosphorylation of the non-catalytic subunit, like in budding yeast (42). In addition, it has been proposed that in human cells, Mus81 is negatively controlled by Wee1 (45). Interestingly, a recent work has shown that, in *S. pombe*, the cell cycle–dependent phosphorylation of Eme1^Mms4^ is Cdc2^CDK1^-dependent but Plo1^PLK1^-independent (46), unlike in budding yeast and human cells, thus showing differences among organisms in the way that Mus81-Eme1/Mms4 is regulated. Moreover, in fission yeast, Eme1^Mms4^ hyperphosphorylation is stimulated when cells are treated with camptothecin or bleomycin. This induced-hyperphosphorylation is Rad3-dependent and increases the activity of *S. pombe* Mus81-Eme1 (46).

Despite the recent findings on Mus81-Eme1/Mms4 regulation and its established function in the resistance to some DNA-damaging agents that hinder chromosome replication, it is still largely unknown how this endonuclease operates when cells need to cope with DNA damage at RFs. Mus81-Eme1/Mms4 is known to be crucial for dealing with DNA lesions such as those induced by the model DNA-damaging agent methyl methanesulfonate (MMS), which interfere with RF progression. Yet, because this endonuclease can cleave DNA RFs, its activity must be carefully controlled somehow under these situations of DNA damage. Although above-mentioned works have shown that Mus81-Mms4 is activated when cells finish S-phase in the absence of damaged DNA (42,43), and in *S. pombe* its activity is enhanced by some DNA-damaging drugs in G2-cells (46), it is currently unclear how Mus81-Mms4 is regulated in response to DNA lesions occurring during replication. Here, we have analysed the role of budding yeast Mus81-Mms4 under conditions of DNA damage during S-phase and show that this endonuclease is necessary for successful chromosome replication when cells are exposed to MMS. However, our data indicate that Mus81-Mms4 activation is not induced by the presence of DNA lesions and that its function to respond to DNA damage, which allows cell survival, is carried out after completion of bulk genome replication.

## MATERIALS AND METHODS

### Strains, media, cell cycle synchronization, cell viability

The yeast strains used in this work were constructed by standard procedures and are listed in Supplementary Table S1. The pYM (47) and the pML (48) plasmid series were used as templates for polymerase chain reaction. Yeast cells were grown at 30°C in YP medium (1% yeast extract, 2% bacto peptone) with 2% glucose. Bacto agar (2%) was added for solid medium. For gene expression using the *GAL1-10* promoter, the YP medium was supplemented with 2% raffinose or 2% galactose. The α-factor mating pheromone was added to a final concentration of 5–10 µg/ml to synchronize cells in G1-phase. HU was used at 0.2 M to block cells in early S-phase. Nocodazole was used at 5 µg/ml to block cells in G2/M. Samples for flow cytometry were collected and processed as described (49), and analysed using a FACSCalibur flow cytometer (BD Biosciences). For fluorescence microscopy analysis, cells were grown in minimal medium supplemented with yeast synthetic drop out (Sigma-Aldrich). Living cells were analysed using an Axiovert200 Zeiss fluorescence microscope with a 63× oil immersion objective. Cell viability was determined by plating cells in triplicate onto YP-glucose (YPD) plates and counting colony-forming units after 3 days of incubation at 30°C.

### Immunoblotting and *in situ* kinase assay

Protein extracts for immunoblotting analysis were prepared from trichloroacetic acid-treated cells as described (50). HA-tagged proteins were detected with the 12CA5 antibody (CBMSO), using horseradish peroxidase–coupled anti-mouse (Vector Labs) as a secondary antibody. Rad53 was detected with the JDI48 antibody (a gift from Dr J. Diffley, Cancer Research UK) and the horseradish peroxidase–coupled protein A (Invitrogen) as a secondary antibody. Immunoreactive bands were visualized using enhanced chemiluminescence (ECL prime, GE Healthcare). The Rad53 *in situ* kinase assay was performed essentially as described (51). For the authophosphorylation reaction, the membranes (Hybond P, GE Healthcare) were incubated for 1 h at room temperature (RT) in kinase buffer containing 10 µCi/ml [γ-^32^P]ATP.

### Drug sensitivity assays

Logarithmic cultures growing at 30°C were normalized to 1 × 10^7^ cells/ml, and 10-fold serial dilutions were spotted onto YP plates containing either glucose or galactose and different concentrations of MMS (Sigma-Aldrich). The plates were incubated at 30°C for 48–72 h.

### Dense isotope transfer

Dense isotope substitution experiments were based on (52) and performed as described (53). The cells were grown for eight generations at 30°C in minimal medium with 0.1% ^13^C glucose and 0.01% ^15^N (NH_4_)_2_SO_4_ (CK Gas Products) (‘heavy medium’). They were then synchronized in G1, transferred to ‘light’ minimal medium [2% ^12^C glucose and 0.01% ^15^N (NH_4_)_2_SO_4_] and released into fresh minimal medium either with or without MMS (0.033%). The DNA was digested with the restriction enzymes *Cla*I and *Sal*I, and the digestion products were separated on CsCl gradients. They were fractionated, and the fractions were blotted and hybridized with specific DNA probes recognizing the *Cla*I/*Sal*I restriction fragments from chromosome VI (54). DNA replication was seen as the transfer from the heavy–heavy (HH) peak (unreplicated DNA), in which the two DNA strands are substituted with dense isotopes, to the heavy–light (HL) peak (replicated DNA), where only the parental strand has the heavy isotopes. The extent of replication was calculated from the following equation: % replication = 100[0.5 HL/(HH + 0.5 HL)].

### Pulse field gel electrophoresis

Genomic DNA samples were obtained from 10^8^ cells, and prepared in low melting point agarose plugs, as described (55). Yeast chromosomes were separated in a 1% agarose-TBE gel by pulsed-field gel electrophoresis (PFGE) at 14°C, using a Gene Navigator System, from Pharmacia Biotech. The electrophoresis were carried out at 180 V (6 V/cm) for 24 h, with 90 s, 105 s and 125 s pulses for 9.6, 6 and 8.4 h, respectively. The gels were stained with ethidium bromide and scanned on a Gel Doc 2000 (Biorad). Quantification of the chromosome bands was performed using the Quantity One program (Biorad).

### Nuclease activity assays

The oligonucleotides used to make the DNA substrates were based on (24). For the construction of a model RF, the oligonucleotides were RF-1: 5′-GACGCTGCCGAATTCTGGCGTTAGGAGATACCGATAAGCTTCGGCTTAAG; RF-2: 5′-ATCGATGTCTCTAGACAGCACGAGCCCTAACGCCAGAATTCGGCAGCGTC; RF-3: 5′-CTTAAGCCGAAGCTTATCGGTATCT and RF-4: 5′-GCTCGTGCTGTCTAGAGACATCGAT. For the construction of a 3′-FL, the oligonucleotides were RF-1, RF-2 and RF-4. For the formation of the synthetic structures used as the substrates in the nuclease assays, the oligonucleotide RF-1 was 5′-^32^P-labelled using [γ-^32^P]ATP (Perkin Elmer) and T4 Polynucleotide kinase (New England Biolabs) and then annealed with an excess of their complementary oligonucleotides. The annealing was carried out by heating the DNA molecules for 10 min at 80°C in 200 mM NaCl plus 60 mM Tris–HCl (pH 7.5) buffer, followed by cooling to RT.

Immunoaffinity purification and nuclease assays were based on a previously described method (56). HA-Mms4 was immunoaffinity purified from 5 × 10^8^ cells, which were disrupted using glass beads in 800 µl of binding buffer (40 mM Tris–HCl, pH 7.5; 100 mM AcK; 4% glycerol; 0.1% NP40; 5 mM DTT; 5 mM NaF; 5 mm Na_4_P_2_O_7_; protease inhibitors cocktail (2×) from Roche—Complete), and cleared by 40 min centrifugation at 4°C, top speed. The supernatant was incubated for 180 min at 4°C with 12CA5 antibody and followed by 60 min incubation with 15 µl of Protein A-Sepharose (Sigma-Aldrich). The Sepharose-bound proteins were centrifuged, washed extensively and used directly for the reactions. The reaction mixtures for the nuclease activity assays (25 µl) contained labelled DNA substrate (40 fmol) in 100 mM NaCl, 50 mM Tris–HCl, pH 8, 3 mM MgCl_2_, 0.5–1 µg poly[dI-dC], DTT 0.2 mM, plus the affinity purified tagged-Mms4. The reactions were incubated for 60 min at 30°C and stopped with denaturing stop buffer (final concentration: 19% formamide, 4 mM EDTA, 0.01% xylene-cyanol and 0.01% bromophenol). The ^32^P-labelled products were analysed by electrophoresis through 12.5% denaturing gels containing 7 M urea. Gels were run at constant 23 W.

## RESULTS

### Mus81-Mms4 is important for the cellular response to DNA damage that originates during S-phase

**Figure 1.**
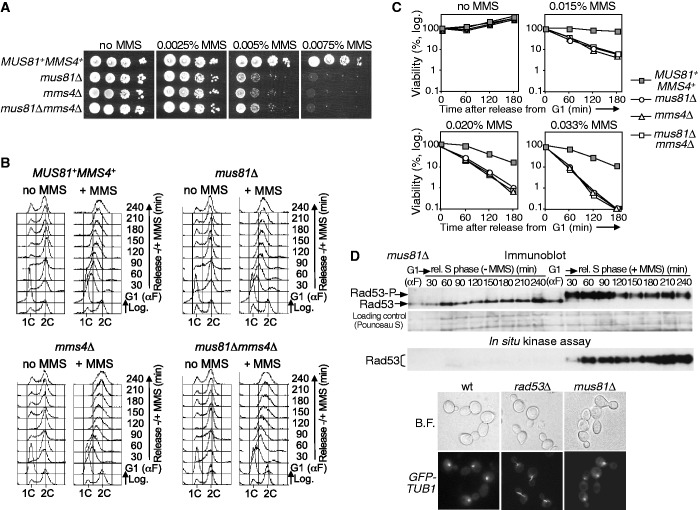
Mus81-Mms4 is necessary to respond to DNA damage occurring during S-phase. (**A**) Sensitivity to MMS. Serial dilutions (10-fold) of normalized log-phase cultures were spotted onto YPD plates containing the indicated amounts of MMS and incubated at 30°C for 48–72 h. *MUS81^+^MMS4^+^* (W303-1a strain); *mus81*Δ (YMV39 strain), *mms4*Δ (YMV48 strain), *mus81*Δ *mms4*Δ (YMV49 strain). (**B**) Cells were synchronized in G1 and released in medium ±MMS (0.033%). The cells were collected at the indicated time points and the DNA content was determined by flow cytometry. (**C**) Cell viability after MMS treatment during S-phase. The different MMS concentrations used are indicated at the top of each panel. (**D**) Checkpoint activation. Upper panel: immunoblot analysis of Rad53. Middle panel: *in situ* autophosphorylation assay for Rad53. Bottom panel: fluorescence microscopy; G1-blocked cells were released into medium with MMS (0.033%) for 2 h and spindle elongation was analysed. Wild type (YJT126 strain); *mus81*Δ *GFP-TUB1* (YMV22 strain); *rad53*Δ *GFP-TUB1* (YJT127 strain).

Cells lacking Mus81-Eme1/Mms4 are sensitive to the treatment with different DNA-damaging agents, like the alkylating compound MMS (13–15). To determine the role of this endonuclease specifically in replication over DNA lesions, we restricted DNA damage to a single S-phase and analysed the consequences of the absence of Mus81-Mms4. We first made null mutants of the genes encoding the subunits of the Mus81-Mms4 complex in budding yeast, *mus81*Δ and *mms4*Δ, as well as a double mutant *mus81*Δ*mms4*Δ. The strains were tested for growth on solid medium with MMS. All mutants grew like wild-type cells in medium without drug but were much more sensitive to MMS than the control as expected (Figure 1A). We next studied S-phase progression in cells treated with MMS and examined its viability throughout the experiment. For this, wild-type and mutant cells were synchronized in G1 using α-factor and then released into fresh medium either without or with MMS, at several concentrations. In all cases, the cells finished replication by 60 min in medium without MMS, as shown by flow cytometry, and progressed later to a new cell cycle (Figure 1B). In contrast, in the presence of MMS (for simplicity, only the results with 0.033% MMS are shown), the cells progressed slowly through S-phase, due to the DNA lesions as shown before (54,57). Flow cytometry shows that the slow S-phase in the three mutants treated with MMS was similar to that of the control strain (Figure 1B), suggesting that bulk genome replication is not affected by the absence of Mus81-Mms4 when the DNA is damaged. However, unlike the wild-type control, all mutants showed a high loss of viability during the experiment when treated with MMS, with no differences among the strains (Figure 1C). Therefore, although apparently S-phase progression is similar in wild-type and *mus81/mms4* cells exposed to MMS, cells lacking Mus81-Mms4 might nevertheless have important chromosome replication defects under these conditions that severely affect cell survival.

To exclude that the loss of viability observed was an indirect effect due to a defective S-phase checkpoint in *mus81/mms4* cells, which could yield similar results (54,57), we monitored checkpoint activation. As the results obtained in the previous experiments with the three mutants were identical, we used from now *mus81*Δ as the mutant lacking the endonuclease. First, we analysed the phosphorylation and kinase activity of the checkpoint protein Rad53 in *mus81*Δ cells during S-phase, in the presence or absence of MMS. The immunoblot (Figure 1D) showed that Rad53 was only hyperphosphorylated when *mus81*Δ cells were treated with MMS. Moreover, Rad53 phosphorylation correlated with the acquisition of kinase activity by this protein, as shown by an *in situ* assay (Figure 1D) that allows measuring its autophosphorylation (51). Second, spindle elongation was analysed in S-phase cells treated with MMS, using *TUB1-GFP*. The data (Figure 1D) show that, like the wild-type control, and unlike *rad53*Δ cells, which cannot restrain mitosis despite incomplete DNA replication, spindle elongation was inhibited in *mus81*Δ cells, indicating that the checkpoint prevents mitosis in this mutant. In summary, cells lacking the endonuclease are checkpoint proficient, and the loss of viability after exposure to MMS during S-phase is due to the absence of Mus81-Mms4 and not to a defective checkpoint response. Additionally, these results indicate that, in the absence of DNA damage, lack of Mus81-Mms4 did not induce detectable checkpoint activation.

### Mus81-Mms4 is necessary for successful completion of chromosome replication under DNA-damaging conditions

The experiments above suggested that cells lacking Mus81-Mms4 might accumulate severe problems during replication in the presence of DNA damage, as they showed a significant loss of viability with respect to the wild-type control despite an apparent similar S-phase progression (Figure 1B and C). Therefore, it was necessary to analyse the dynamics of chromosomal replication using higher resolution approaches than flow cytometry.

**Figure 2.**
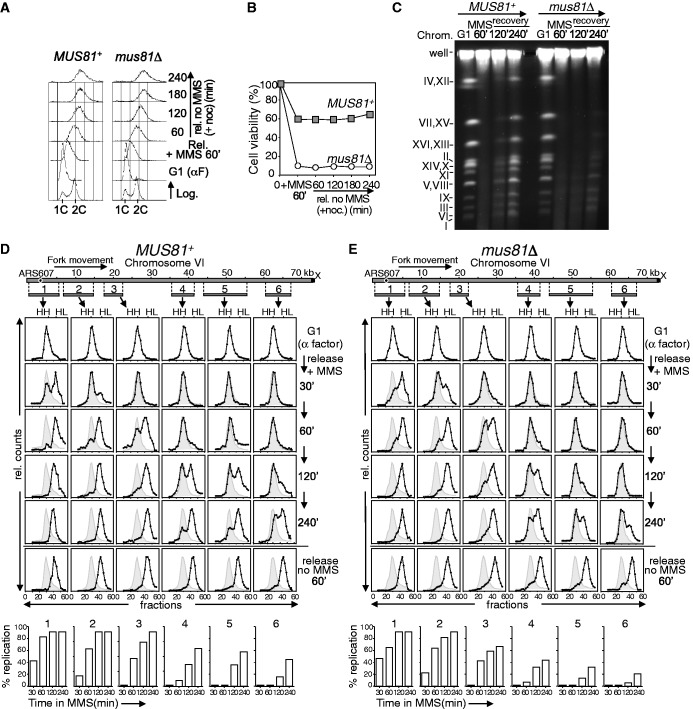
Mus81-Mms4 is required for successful chromosome replication in the presence of DNA damage. (**A–C**) PFGE analysis. *MUS81^+^* (W303-1a strain) and *mus81*Δ (YMV39 strain) cells were treated in S-phase with MMS (0.033%) for 60 min. The MMS was then removed and the cells progressed through S-phase in medium with nocodozale. (**A**) Cell cycle progression was monitored by flow cytometry. (**B**) Cell viability throughout the experiment. (**C**) Ethidium-bromide–stained pulse-field gel. Chromosomes are labelled with Roman numerals. (**D–E**) Density transfer analysis. *MUS81^+^* (YJT110 strain) and *mus81*Δ (YMV17 strain) cells were blocked in G1 in medium with heavy isotopes and released then in medium with light isotopes, ±MMS (0.033%). RF progression in *MUS81*^+^ (**D**) and *mus81*Δ (**E**) cells was followed in a replicon of chromosome VI, using DNA probes recognizing the *Cla*I/*Sal*I fragments 1–6. Probe numbers correspond to fragment numbers. The relative amounts of radioactivity in the hybridized DNA are plotted against the gradient fraction number. The positions of unreplicated (HH) and fully replicated (HL) peaks are indicated. The position of the initial HH peak is shown for comparison (grey area). Quantification of replication in the presence of MMS for every fragment and time point is shown at the bottom.

We first studied the role of this endonuclease in the replication of damaged DNA by analysing chromosomes by PFGE, after treating cells with MMS (Figure 2A–C). *MUS81*^+^ and *mus81*Δ cells were synchronized in G1 with α factor and then released into S-phase in medium containing MMS. After 60 min, the MMS was removed and the cells were released into fresh medium and allowed to progress through S-phase. Nocodazole was added to avoid progression through a new cell cycle, stopping cells at G2/M. Flow cytometry (Figure 2A) showed that after 60 min with MMS, both *MUS81*^+^ and *mus81*Δ cells remained with less than 2 C DNA content. After removing the MMS, both strains progressed similarly through S-phase and reached the 2 C DNA peak, indicating completion of bulk DNA replication. Consistent with the results in Figure 1B and C, the viability of *MUS81*^+^ cells was high throughout the experiment, whereas it was reduced in the *mus81*Δ mutant (Figure 2B).

The PFGE technique resolves linear chromosomes from agarose-embedded cells, but DNA containing replication bubbles remains trapped in the loading wells (58). In both *MUS81*^+^ and *mus81*Δ cells, intact chromosomal DNA from G1-arrested cells was separated as discrete bands. After 60 min MMS treatment in S-phase, no chromosome bands were detected, indicating ongoing replication in both strains (Figure 2C), consistent with flow cytometry (Figure 2A). In *MUS81*^+^ cells, full-length chromosomes started re-entering the gel after 2 h recovery in MMS-free medium, and by 4 h a high proportion of chromosomal DNA appeared as clear bands (about 80% with respect to the G1-signal), indicating that in most cells the chromosomes were recovered from the MMS-induced lesions and completed replication. On the contrary, in *mus81*Δ cells, most of the chromosomal DNA was retained in the wells 4 h after recovery from MMS treatment (only 15% with respect to the G1-signal appeared as discrete bands), showing that it was not completely replicated. Thus, these results indicate that Mus81-Mms4 is required for successful chromosome replication when the DNA is damaged, even when S-phase progression seems to be completed in both wild-type and *mus81*Δ cells (Figure 2A), which in turn explains why cells lacking the endonuclease lose viability under these conditions (Figures 1C and 2B). These data are in agreement with a previous report showing similar results (30), where it was proposed a function for Mus81 in resolving stalled forks or recombination intermediates after DNA damage to allow DNA synthesis.

Although the PFGE data indicate a role for Mus81 in facilitating the completion of replication under DNA-damaging conditions, this technique does not show whether/how fork progression is affected and does not allow an estimation of the extent of the replication defects. To study replication in more detail, we used a second approach based on dense isotope transfer, which provides a method to study ongoing DNA synthesis at a particular replicon by analysing the progression of DNA RFs (52,59,60). Wild-type and *mus81*Δ cells were grown in medium with ‘heavy’ isotopes, synchronized in G1 and released into medium containing ‘light’ isotopes, either with or without MMS. The replication of six DNA restriction fragments was analysed in a replicon of 75 kb of chromosome VI (54), from the early origin *ARS607* to the end of the chromosome (Figure 2D–E). In both *MUS81*^+^ (Figure 2D) and *mus81*Δ (Figure 2E) strains, all DNA fragments were in the HH (unreplicated DNA) peak in G1-cells (top row). In both strains, the fragments shifted to the HL (replicated DNA) peak by 60 min after release from G1 medium without MMS (bottom row), which indicates that they had been replicated. When *MUS81*^+^ cells were released into S-phase in medium with MMS, the fragment 1 containing *ARS607* largely shifted to the HL position by 30 min, and at this time, there was some HL DNA in fragment 2 (Figure 2D). Replication progressed rightward: fragment 3 shifted to the HL peak by 60 min and the replication of fragments 4–6 proceeded progressively at later time points (the data are quantified at the bottom of Figure 2D). Thus, as previously described (54,61), DNA RFs move slowly but efficiently through a damaged replicon.

In *mus81*Δ cells, the fragment 1 also shifted to the HL peak by 30 min after release from G1 in medium with MMS (Figure 2E), indicating that the firing of *ARS607* occurs like in the wild-type control. Moreover, replication progressed rightward, as seen by the shifting of the fragments to the HL peak. However, the estimation of the extent of replication along the replicon indicated that chromosome replication was compromised in the absence of Mus81. Thus, at 120 min, the percentage of replicated (HL) DNA in fragments 3–6 was reduced with respect to the wild type. For example, at this time point, the number of forks that had passed completely fragments 5–6 in the *mus81*Δ mutant was about three times less than in the control strain (quantification is shown at the bottom of Figure 2D–E). Later, at 240 min, only 43, 30 and 20% of forks in *mus81*Δ cells had passed completely fragments 4, 5 and 6, respectively, showing defects in the replication of the chromosome when compared with the *MUS81*^+^ strain, in which 63, 57 and 44% of forks passed the same DNA fragments. Therefore, although bulk DNA replication seems similar in *MUS81*^+^ and *mus81*Δ cells (Figures 1B and 2A), more sensitive assays indicate that chromosome replication in the presence of DNA damage is perturbed in cells lacking Mus81-Mms4. The dense isotope substitution experiments also show that the absence of this endonuclease does not cause extensive fork collapse or persistent fork stalling when cells are treated with MMS. In fact, forks progress considerably along the studied replicon before clear chromosomal replication defects are observed at late time points, when DNA lesions accumulate due to the continued exposure to MMS. Nevertheless, these replication problems due to the absence of Mus81 are important enough to account for the incompletion of chromosome replication observed by PFGE (Figure 2C) as well as for the reduced viability of *mus81*Δ cells under the assayed DNA-damaging conditions (Figures 1C and 2B).

### Mus81-Mms4 is not required to resume DNA replication after replicative stress caused by acute treatment with HU

We next asked whether Mus81-Mms4 is also necessary to cope with other kinds of DNA replication perturbations that, like MMS, cause RF stalling, but unlike this drug, not necessarily DNA damage. Thus, we studied the possible involvement of this endonuclease in replication resumption after replicative stress originated in a single S-phase by the ribonucleotide reductase inhibitor HU. There are some apparently contradictory data on the role of Mus81-Eme1/Mms4 in facilitating DNA replication after fork stalling caused for HU. For example, mammalian Mus81 causes double-strand breaks (DSB) after prolonged exposure to replicative stress induced by HU (62,63), and a role for this protein in RF restart after HU treatment has been proposed (62). However, while mouse ES cells lacking Mus81 increase their sensitivity to HU (62), MUS81-depleted U2OS cells exhibit increased resistance to HU (63). On the other hand, although *S. pombe* and *S. cerevisiae* cells lacking Mus81 show sensitivity to chronic treatment with HU (13,29,30), *S. pombe mus81*Δ cells are not significantly sensitive to acute exposure to HU (17), and Mus81 does not have an apparent role in the resumption of DNA replication after treatment with this drug in fission yeast (17). These diverse data could likely reflect distinct biological responses in different systems to the same challenge, or could be the consequence of different experimental conditions that lead to different results. Therefore, we decided to analyse the requirement of budding yeast Mus81-Mms4 for the completion of chromosome replication after early S-phase block caused by acute treatment with HU.

**Figure 3.**
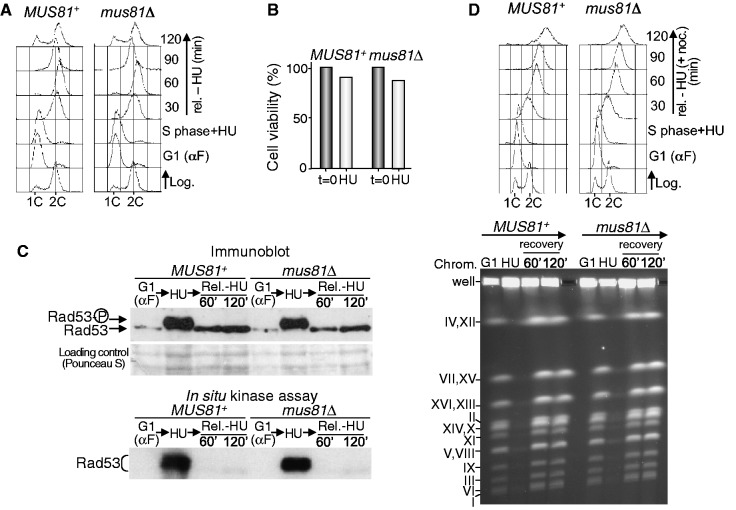
Mus81-Mms4 is not required to resume replication after an HU block. (**A**) *MUS81^+^* (W303-1a strain) and *mus81*Δ (YMV39 strain) cells were blocked in G1, released into medium with 0.2 M HU for 90 min and then allowed to progress through the cell cycle in medium without the drug. Samples were taken at the indicated time points and the DNA content was analysed by flow cytometry. (**B**) Cell viability after HU treatment. (**C**) Immunoblot analysis of Rad53 (upper panel) and *in situ* kinase assay for Rad53 (bottom panel). (**D**) PFGE analysis. The cells were blocked in G1, released into medium containing 0.2 M HU for 90 min and then released from the HU block in medium with nocodazole. Cell cycle progression was monitored by flow cytometry (upper panel). The ethidium-bromide–stained pulse-field gel is shown at the bottom. Chromosomes are labelled with Roman numerals.

*MUS81^+^* and *mus81*Δ cells were arrested in G1 with α factor and then released into S-phase in medium containing 0.2 M HU. It is well established that under the assayed conditions, DNA replication initiates from early origins and forks stall within 10 kb from an origin (64). After 90 min, the cells were released into medium without HU and allowed to progress through S-phase. Flow cytometry (Figure 3A) shows that HU-blocked cells remained with a DNA content close to 1C. More than 95% of these cells were budded, confirming release from G1. After removing HU, *MUS81^+^* and *mus81*Δ cells progressed through S-phase, reached the 2C DNA peak similarly and started a new cell cycle, indicating that DNA replication and mitosis proceeded normally. Moreover, both control and mutant cells maintained high viability after HU treatment (Figure 3B). These results suggest that the Mus81-Mms4 endonuclease is not necessary for DNA resumption after blocking forks with HU. The same results were obtained even if the HU treatment was extended (Supplementary Figure S1). To discard that the completion of DNA replication after HU block in the absence of Mus81 was just due to new replication initiation events that could rescue remaining stalled forks, we monitored the activation of the Rad53 checkpoint protein (Figure 3C). As shown in the immunoblot, Rad53 appeared hyperphosphorylated when *MUS81^+^* and *mus81*Δ cells were treated with HU, which correlated with its kinase activity (Figure 3C). In both cases, when HU was removed, Rad53 became dephosphorylated and, consistently, no kinase activity was observed. If there were remaining blocked or collapsed forks owing to the absence of Mus81-Mms4 after HU treatment, the checkpoint would continue to be activated, which, in turn, would block new replication initiation from late replication origins (64). However, this was not the case, as Rad53 did not remain activated once HU was removed. Therefore, although new replication initiation is expected to occur in the experiment, this result reinforces the data above indicating that the absence of Mus81-Mms4 does not impede normal fork resumption after HU treatment.

To show more precisely that Mus81-Mms4 was not required for the response to fork stalling caused by HU, we analysed chromosomes by PFGE after treating *MUS81^+^* and *mus81*Δ cells with this drug. Both strains were blocked in G1 and then released into S-phase in medium with HU, which was subsequently removed. Nocodazole was added to stop cells in G2/M. Flow cytometry (Figure 3D, upper panel) showed that both control and mutant cells reached a 2C DNA content after release from the HU block, indicating that bulk DNA synthesis had finished. PFGE (Figure 3D, lower panel) showed that intact chromosomes from G1-blocked cells were separated as discrete bands. In both strains, chromosomal DNA did not enter the gel after HU treatment, indicating ongoing replication. In both *MUS81*^+^ and *mus81*Δ cells, the chromosomes re-entered the gel after 60 min recovery in medium without HU and appeared as discrete bands with no differences between strains, showing that they recovered from the HU block and finished replication.

Thus, completely different to the situation in which the DNA is damaged with MMS, Mus81-Mms4 is not required for the completion of chromosome replication when RFs stall due to acute treatment with HU. This strongly suggests that under these conditions, at least in budding yeast, Mus81-Mms4 is not involved in the cleavage of stalled forks that could facilitate recombination-dependent fork restart.

### Mus81-Mms4 activation occurs when cells finish S-phase, even in the presence of DNA lesions that interfere with chromosome replication

The results presented in this work indicate that Mus81-Mms4 is necessary for chromosome replication under DNA-damaging conditions. At first glance, this might seem paradoxical, as the regulation of Mus81-Mms4 through Mms4 phosphorylation restrains its normal function to a narrow period of the cell cycle, just before chromosome segregation, and maintains the nuclease activity low during S-phase (42,43). However, Mus81-Mms4 regulation was previously only studied in an unperturbed cell cycle, in the absence of DNA damage. This raises the question of whether DNA lesions that interfere with chromosome replication and reduce cell survival in the absence of Mus81-Mms4, can induce or modify somehow the regulation of this endonuclease.

**Figure 4.**
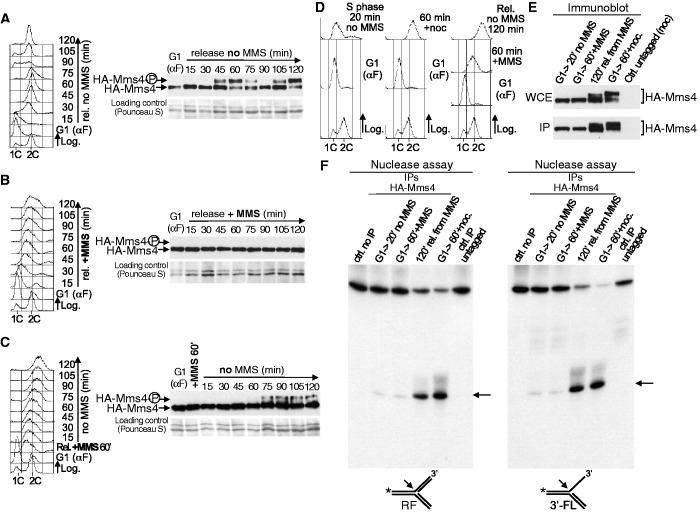
Mus81-Mms4 activation is not induced by DNA damage and occurs when cells finish S-phase. (**A**) *HA-MMS4* cells (YMV33 strain) were synchronized in G1 and then released into fresh medium. Cell cycle progression was monitored by flow cytometry (left). Mms4 phosphorylation was analysed by immunoblot (right). (**B**) *HA-MMS4* cells were blocked in G1 and then released into medium with MMS (0.02%). Left: Flow cytometry. Right: Immunoblot analysis of Mms4. (**C**) *HA-MMS4* cells were synchronized in G1 and then released into S-phase in medium with MMS (0.02%) for 60 min. The MMS was then removed and the cells were allowed to progress through S-phase. Left: Flow cytometry. Right: Immunoblot analysis of Mms4. (**D**) Cell cycle experiments for the nuclease assays. *HA-MMS4* cells were blocked in G1 and the culture was then divided in three. One culture was released in medium without MMS for 20 min; another one in medium without MMS plus nocodazole, for 60 min; the third one was released into S-phase for 60 min in medium with MMS (0.02%). The MMS was then removed and the cells were allowed to progress through the cell cycle. Cell cycle progression was followed by flow cytometry. (**E**) The extracts were prepared from cells taken at the indicated time points and HA-Mms4 was immunoaffinity purified. Mms4 was analysed by immunoblot in the whole cell extracts (WCE) and the yield of the IP was estimated. About 2% of the total amount of the IP protein used for the nuclease assays was loaded. (**F**) The nuclease activity was assayed by the resolution of a model RF (left panel) and a 3′-FL (right panel). An arrow indicates the labelled product resulting from the cleavage of each substrate. The controls were a reaction without extract and a nuclease assay using IP-extracts from untagged cells blocked in G2/M.

To answer this question, we first analysed Mms4 phosphorylation in the presence of DNA damage and compared it with its previously known modification in an unperturbed cell cycle (Figure 4A–C). *HA-MMS4* cells were synchronized in G1 and then released in medium either without or with MMS (Figure 4A–C). The cells progressed through S-phase and finished the cell cycle in medium without MMS (Figure 4A). The immunoblot (Figure 4A) shows that Mms4 underwent cell cycle–dependent phosphorylation, as shown before (42,43). Thus, Mms4 was hyperphosphorylated at 45 min after release from G1, when the cells reached a 2C DNA content, as shown by an electrophoretic mobility shift, and became dephosphorylated later, when they progressed through a new cell cycle. However, in medium with MMS (Figure 4B), Mms4 did not present changes detectable by immunoblotting, even after 120 min in the presence of MMS, a treatment that would have caused a reduction in cell viability and affected DNA replication in the absence of Mus81-Mms4 (Figures 1 and 2). The lack of visible Mms4 phosphorylation suggests that Mus81-Mms4 remained with low activity during S-phase, despite the presence of DNA damage. Finally, cells were released from G1-arrest in medium with MMS for 60 min, and the MMS was then removed so that the culture could recover and progress through the cell cycle (Figure 4C). Phosphorylation of Mms4 was detected 75 min after removing the MMS, when most cells had a 2C DNA content (Figure 4C), suggesting that Mus81-Mms4 becomes active only at the end of S-phase or in G2/M, despite the previous presence of damaged DNA. Together, these results indicate that Mms4 phosphorylation is not induced by DNA damage and that only occurs when cells finish S-phase.

To confirm directly that DNA lesions during S-phase do not induce Mus81-Mms4 activation, we performed nuclease assays (Figure 4D–F). An *HA-MMS4* culture was blocked in G1 and split into three parts: one part was released in medium without MMS for 20 min (cells in S-phase, no DNA damage), another one in medium without MMS but containing nocodazole for 60 min (cells in G2/M, no DNA damage) and the third one in medium with MMS for 60 min (cells in S-phase, DNA damage). In the latter, the MMS was subsequently removed and the cells were allowed to progress for 120 min to reach the 2C DNA content. All cultures were monitored by flow cytometry (Figure 4D) and budding index estimation. As shown in the immunoblot (Figure 4E), Mms4 was not hyperphosphorylated in cells in S-phase, regardless of having been treated with MMS, in agreement with data in Figure 4A–C. However, when MMS-treated cells were released from the drug and allowed to reach the end of S-phase, Mms4 was hyperphosphorylated, consistent with Figure 4C, and like in G2/M-blocked cells that were used as a positive control. In each case, HA-Mms4 was immunoprecipitated from the cell extracts (Figure 4E) and the nuclease activity was tested (Figure 4F). For the assays, we used two different ^32^P-labelled synthetic structures that are substrates of Mus81-Mms4 and are generated during DNA replication or DNA replication-associated repair: a model RF and a 3′-FL. Mms4 is a non-catalytic subunit, but it was shown that the immunoprecipitation (IP) of Mms4 from extracts yields nuclease activity and, therefore, the Mus81-Mms4 complex immunoprecipitates and is functional under these conditions (65). Figure 4F shows that the nuclease activity of Mus81-Mms4, determined by the appearance of clear labelled products, was only high when Mms4 was immunoprecipitated from extracts obtained from cells that had finished S-phase after MMS treatment. This activity was similar to that of Mus81-Mms4 taken from G2/M arrested cells that had not been treated previously with DNA damage. However, when Mms4 was immunoprecipitated from S-phase cells treated with MMS, or from untreated S-phase cells, the nuclease activity was low. These results show that, although Mus81-Mms4 is necessary for cells to cope with DNA lesions originated during S-phase by MMS, DNA damage does not induce the activation of the nuclease, whose full activity is restrained until cells finish bulk genome replication, like in an unperturbed cell cycle.

### The absence of Sgs1 or Yen1 does not have an effect on Mus81-Mms4 regulation

As mentioned previously, Mus81-Mms4 functionally overlaps with Sgs1-Top3-Rmi1 and is required for cell viability in the absence of this RecQ-helicase complex (13,15,24–26). It is possible that most common substrates for Mus81-Mms4 and Sgs1-Top3-Rmi1 are resolved by dissolution by the latter, but that they need to be processed by Mus81 when the RecQ complex is not functional. In fact, some DNA intermediates that persist after MMS treatment in Rmi1-deficient cells or in the absence of Sgs1 can be removed by Mus81-Mms4 (66,44). Likewise, the sensitivity of *sgs1*Δ cells to some DNA-damaging agents like MMS increases when Mus81-Mms4 is not fully active (43). Mus81-Mms4 has also overlapping functions with the Yen1 resolvase during DNA repair, and cells lacking both Mus81 and Yen1 show hypersensitivity to DNA-damaging drugs (29–32). Due to these functional links, we asked whether the absence of Sgs1-Top3-Rmi1 and Yen1 has an effect on Mus81-Mms4 regulation, as it was possible that this endonuclease could be required under these pathological circumstances at times at which normally is not.

**Figure 5.**
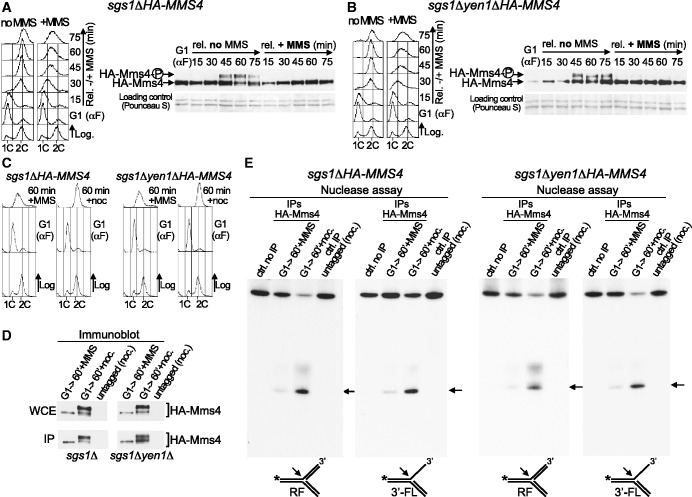
Mus81-Mms4 regulation is not modified in cells lacking Sgs1 or Yen1. (**A**) *sgs1*Δ*HA-MMS4* cells (YMG21 strain) were synchronized in G1 and then released into fresh medium ±MMS (0.02%). Cell cycle progression was monitored by flow cytometry (left). Immunoblot analysis of Mms4 (right). (**B**) G1-blocked *sgs1*Δ*yen1*Δ*HA-MMS4* cells (YSG56 strain) were released into fresh medium ±MMS (0.02%). Left: Flow cytometry. Right: Immunoblot analysis of Mms4. (**C**) Cell cycle experiments for the nuclease assays. Cells were synchronized in G1 and released in medium with MMS (0.02%) for 60 min or in medium without MMS (plus nocodazole). (**D**) The extracts were prepared from cells taken at the indicated time points and HA-Mms4 was immunoaffinity purified. Mms4 was analysed by immunoblot in the whole cell extracts (WCE), and the yield of the IP was estimated. About 2% of the total amount of the IP protein used for the nuclease assays was loaded. (**E**) The nuclease activity was assayed by the resolution of a model RF and a 3′-FL. The arrows indicate the labelled products resulting from the cleavage of each substrate. The controls were a reaction without extract and a nuclease assay using IP-extracts from untagged cells blocked in G2/M.

To address this question, *sgs1*Δ*HA-MMS4* cells were blocked in G1 and released into medium without or with MMS (Figure 5A). Flow cytometry indicates that these cells progressed normally through S-phase in the absence of the drug and slowly when exposed to MMS. The immunoblot shows that, in the absence of MMS, the cell cycle–dependent phosphorylation of Mms4 was like in *SGS1*^+^ cells (Figure 4A). Likewise, like in *SGS1*^+^ cells (Figure 4B), Mms4 did not show an electrophoretic mobility shift when the *sgs1*Δ cells were treated with MMS in S-phase. We also analysed Mms4 phosphorylation under the same conditions in *sgs1*Δ*yen1*Δ*HA-MMS4* cells (Figure 5B). G1-blocked cells were released into medium without or with MMS. These cells progressed in S-phase similarly to *SGS1*^+^*YEN1*^+^ (Figure 4A and B) and *sgs1*Δ cells (Figure 5A), both in the absence or the presence of MMS. The immunoblot (Figure 5B) shows that when the *sgs1*Δ*yen1*Δ cells were not treated with MMS, Mms4 phosphorylation occurred as in *SGS1*^+^*YEN1*^+^ cells (Figure 4A), whereas Mms4 did not present electrophoretic mobility changes indicative of phosphorylation when the cells were exposed to MMS in S-phase. These results indicate that the absence of Sgs1 or Yen1 does not cause changes in the dynamics of activation of this endonuclease, even in the presence of DNA damage during chromosome replication. Moreover, these data also show that the hyperphosphorylation of Mms4 does not increase in budding yeast cells lacking Sgs1 with respect to *SGS1*^+^ cells (compare Figures 4A and 5A), unlike Eme1^Mms4^ phosphorylation in *S. pombe* (46). Mms4 phosphorylation does not increase either in the absence of both Sgs1 and Yen1 (compare Figures 4A and 5B).

Next, to confirm the above data, we assayed Mus81-Mms4 nuclease activity (Figure 5C–E). The *sgs1*Δ*HA-MMS4* and *sgs1*Δ*yen1*Δ*HA-MMS4* cells were arrested in G1; the cells were then released from the block for 60 min, either in the presence of MMS (cells in S-phase, DNA damage) or in medium without MMS plus nocodazole (cells in G2/M, no DNA damage). The cultures were monitored by flow cytometry (Figure 5C) and microscopy. The immunoblots (Figure 5D) show that Mms4 was not hyperphosphorylated in the extracts of S-phase cells treated with MMS, in agreement with data in Figure 5A and B. On the contrary, Mms4 was hyperphosphorylated in both strains in cells in G2/M. In each case, HA-Mms4 was immunoprecipitated from the cell extracts (Figure 5D), and the nuclease activity was analysed. Figure 5E shows that in *sgs1*Δ and *sgs1*Δ*yen1*Δ strains, the nuclease activity of Mus81-Mms4 was low when the complex was immunoprecipitated from S-phase cells treated with MMS. However, when Mms4 was immunoprecipitated from G2/M-cells, Mus81-Mms4 activity was more robust, as shown by the appearance of significant amount of labelled products from the substrates used. Therefore, the mode of regulation of Mus81-Mms4 is not apparently altered when cells lack Sgs1 or Yen1, even in the presence of damaged DNA during S-phase. Thus, even when the function of Mus81-Mms4 becomes more important to respond to DNA damage due to the absence of Sgs1 (43), the full activity of the endonuclease is still prevented until bulk DNA synthesis is completed.

### Mus81-Mms4 functions after completion of bulk DNA replication to respond to DNA damage present in S-phase

**Figure 6.**
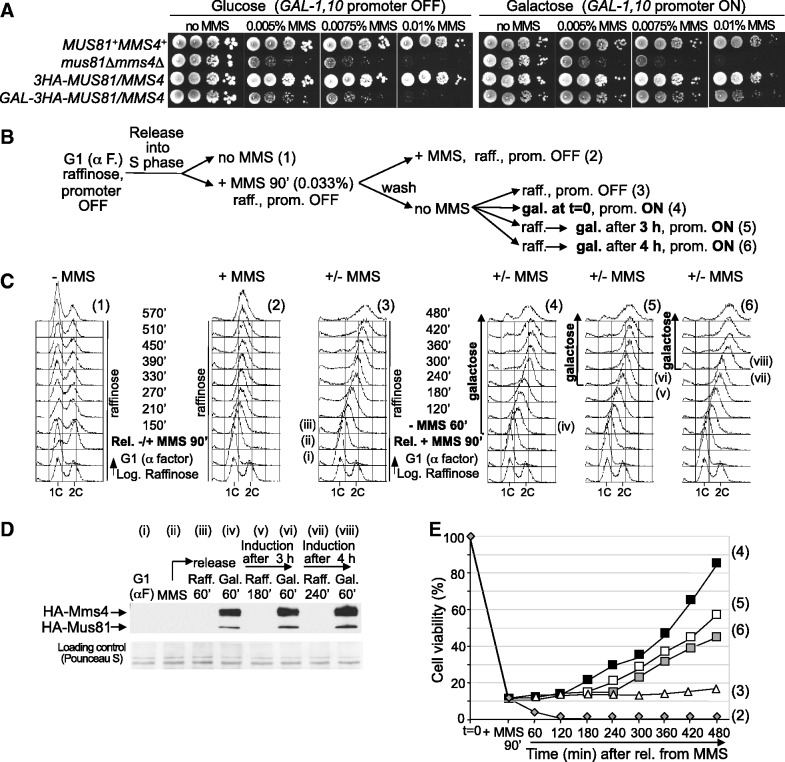
Mus81-Mms4 functions after bulk DNA synthesis to respond to DNA damage originated in S-phase. (**A**) Sensitivity to MMS. Serial dilutions (10-fold) of normalized log-phase cultures were spotted onto YP-Glucose or YP-Galactose plates containing the indicated amounts of MMS and incubated at 30°C for 48–72 h. *MUS81^+^MMS4^+^* (W303-1a strain); *mus81*Δ *mms4*Δ (YMV49 strain); *3HA-MUS81*, *3HA-MMS4* (YSG24 strain); *GAL-3HA-MUS81*, *GAL-3HA-MMS4* (YSG23 strain). (**B**) Scheme of the experiment. Numbers on the right correspond to those on the flow cytometry panels and in the graphic of cell viability. (**C**) Cell cycle progression was followed by flow cytometry. Roman numerals indicate the time points at which protein samples were taken for immunoblot analysis (**D**). (**E**) Cell viability analysis.

As Mus81-Mms4 activation occurs only at the end of S-phase despite the previous originated DNA damage, we reasoned that the problems caused by MMS during replication in the absence of this endonuclease should be reversed by new expression of the complex before the cells were plated for viability analysis. To test this hypothesis, we made a strain in which the endogenous *MUS81* and *MMS4* were placed under control of the *GAL1-10* promoter to allow their conditional expression. To characterize this strain, the sensitivity of *P_GAL1-10_-MUS81 P_GAL1-10_-MMS4* cells to MMS was analysed in medium with glucose or galactose. Figure 6A shows that these cells grew normally in medium without MMS and behaved like a *mus81*Δ*mms4*Δ mutant in medium with MMS and glucose (*GAL-1,10* promoter OFF). However, in medium with galactose (*GAL-1,10* promoter ON), these cells increased notably their resistance to MMS with respect to *mus81*Δ*mms4*Δ. This result indicates that the induced expression of Mus81 and Mms4 in the same strain reconstitutes a functional complex, at least to an extent that allows our experimental approach.

To analyse the reversibility of the problems caused by MMS in S-phase cells lacking Mus81-Mms4, *P_GAL1-10_-MUS81 P_GAL1-10_-MMS4* cells were grown in medium with raffinose (*GAL1-10* promoter inactive), synchronized in G1 and then released into medium containing raffinose, either without or with MMS. After 90 min, the culture containing MMS was divided: one part was held in medium with raffinose plus MMS; in the others, the MMS was removed and the cells were transferred to medium either with raffinose or with galactose. The induction with galactose was also carried out 3 and 4 h after eliminating the MMS (a scheme of the experiment is drawn in Figure 6B). Cell cycle progression was followed by flow cytometry (Figure 6C) and the expression of Mus81 and Mms4 was monitored by immunoblot (Figure 6D). Cell viability was estimated at the indicated time points (Figure 6E).

Cells lacking Mus81 and Mms4 progressed normally through several cell cycles after release from the G1 block in medium without MMS (Figure 6C, panel 1). In contrast, these cells progressed slowly through S-phase in the presence of MMS (Figure 6C, panel 2), which was accompanied by a high loss of viability (Figure 6E). Thus, *P_GAL1-10_-MUS81 P_GAL1-10_-MMS4* cells, under repressive conditions for the *GAL1-10* promoter, behaved similarly to a *mus81*Δ*mms4*Δ strain in a medium with glucose (Figure 1). After MMS removal in the continued absence of Mus81 and Mms4, the cells progressed through S-phase and reached the 2C DNA content (Figure 6C, panel 3). However, the cells did not recover viability and, even 8 h after removing the MMS, did not complete the cell cycle. When *P_GAL1-10_-MUS81 P_GAL1-10_-MMS4* cells were transferred to galactose medium lacking MMS (Figure 6C, panel 4), Mus81 and Mms4 were induced within an hour (Figure 6D). At different times, on release into galactose medium lacking MMS, cell viability was monitored on YPGal medium (Supplementary Figure S2A), or on YPD medium that repressed expression of Mus81 and Mms4 once again (Mus81 and Mms4 were degraded rapidly under such conditions, as shown in Supplementary Figure S2B). Importantly, the viability was only restored when Mus81 and Mms4 were expressed several hours after release from MMS, around the time that cells reached the end of S-phase (Figure 6C, panel 4). This strongly suggests that Mus81-Mms4 only carries out its function in the response to DNA damage after bulk DNA synthesis.

To further demonstrate that Mus81-Mms4 works after bulk DNA replication to respond to DNA damage present in S-phase, its expression was induced when the cells had a 2C DNA content, rather than just after eliminating the MMS. Thus, 3 and 4 h after MMS removal (Figure 6C, panels 5 and 6, respectively), part of the culture growing in raffinose medium was transferred to medium with galactose. The immunoblot (Figure 6D) shows that both subunits were expressed within an hour. Flow cytometry (Figure 6C) and microscopy analysis indicated that in both cases cells finished S-phase and started dividing. Regardless of the time of Mus81-Mms4 induction after MMS removal, cell viability was significantly restored (Figure 6E). Moreover, the recovery of viability occurred progressively but started as soon as the expression of Mus81-Mms4 was induced, unlike the case when it was expressed just after eliminating the MMS. Likely, this is because now the cells were already at the end of S-phase, when Mus81-Mms4 executes its function to respond to DNA damage according to our data.

Together, these results indicate that the consequences of the absence of the Mus81-Mms4 endonuclease during replication of a damaged DNA template are largely reversible by new expression of this complex. Furthermore, these data show that Mus81-Mms4 operates after bulk DNA synthesis to allow cells to cope with DNA damage that originated during chromosomal replication, consistent with the results shown in Figures 4 and 5. It thus appears that Mus81-Mms4 function is temporally separable from bulk genome replication.

## DISCUSSION

We have shown in this work that the structure-specific Mus81-Mms4 endonuclease has a key role in the successful completion of chromosome replication under conditions of DNA damage. Furthermore, we have found that this crucial function for cell survival is strictly controlled, and that Mus81-Mms4 responds to DNA lesions that occur during S-phase and hinder RF progression after the bulk of genome replication has been finished.

Our data indicate that the problems for finishing replication in DNA-damaged cells lacking Mus81-Mms4 are not due to massive fork collapse or lasting and extended fork stalling. Instead, they more likely reflect the result of relatively infrequent events. Nevertheless, these are qualitatively important, as they account for the incompletion of chromosome replication and the high loss of viability in *mus81* mutants after treatment with a DNA-damaging agent like MMS in a single S-phase.

Our results also show that budding yeast Mus81-Mms4 is not required for coping with every kind of perturbation that causes fork block. We have shown that Mus81-deficient cells resume DNA replication normally after fork stalling originated by HU, without loss of viability. Therefore, it is unlikely that, at least in *S. cerevisiae*, Mus81-Mms4 is involved in the cleavage of stalled forks to initiate recombination-dependent fork restart. This is consistent with the fact that recombination is not necessary to complete S-phase after HU arrest (67). Furthermore, in agreement with these data, Mus81-Mms4 shows low activity in cells blocked with HU (43), and in *S. pombe*, Mus81 is displaced from chromatin after HU treatment in a Cds1-dependent manner (17). These results are apparently in contradiction with data from mammalian cells, showing that Mus81-Eme1 cleaves stalled forks following HU exposure (62,63), which is proposed to allow fork restart (62). The different results may reflect distinct responses to stalled RFs between yeast and higher eukaryotes. They may also likely indicate differences between an acute, short HU treatment in our experiments and prolonged HU exposure in those with mammalian cells. In fact, long, but not short, HU treatment can increase the amount of DNA damage and fork-associated DSB (68,69). Mus81-dependent fork cleavage after HU exposure has also been found under pathological situations (70–72), but these are not present in our experimental approach.

MMS causes DNA lesions that trigger fork pausing (54,61,73,74). The fact that Mus81-Mms4 is not required for restart after fork stalling caused by HU makes also unlikely that its role to respond to MMS-induced lesions during replication involves fork cleavage. Instead, it is more probable a function for Mus81-Mms4 in processing DNA intermediates that originate during DNA replication or DNA replication-associated repair, the resolution of which is necessary for finishing chromosome duplication. Several pathways, including base excision repair, homologous recombination and post-replication repair, are required for replication through DNA lesions produced by MMS (61). These pathways generate potential Mus81-Mms4 substrates during the removal of DNA damage, some of which need probably to be resolved at some point by this endonuclease to allow completion of DNA repair and chromosome replication.

The data presented in this work indicate that DNA lesions produced during S-phase do not modify the mode of activation of Mus81-Mms4 that occurs in a normal cell cycle. Mms4 phosphorylation and acquisition of full endonuclease activity in cells treated with MMS during S-phase remain restrained until they finish bulk genome replication, and therefore the regulation of Mus81-Mms4 under DNA-damaging conditions that interfere with DNA replication is like in an unperturbed cell cycle (42,43). Moreover, the absence of the RecQ-helicase Sgs1 or the Yen1 resolvase, proteins with which Mus81-Mms4 functionally interacts, does not have an apparent effect on Mus81-Mms4 regulation. Therefore, the regulation of Mus81-Mms4 through Mms4 phosphorylation is independent of the presence of DNA damage during chromosome replication, even if the number of DNA lesions could accumulate due to pathological situations originated by the absence of proteins with which it has overlapping functions. A recent work has shown that, in *S. pombe*, Eme1^Mms4^ phosphorylation is increased in G2 by damage induced by camptothecin or bleomycin, which is accompanied by enhanced activity of Mus81-Eme1 (46). However, our work indicates that MMS does not induce or modify the phosphorylation of budding yeast Mms4 and therefore the activity of Mus81-Mms4. Furthermore, in budding yeast, the treatment with other DNA-damaging drugs during S-phase or in G2/M does not have a detectable effect on the cell cycle–dependent phosphorylation of Mms4 (Supplementary Figure S3). These results, together with the fact that Eme1-phosphorylation in *S. pombe* is Plk1-independent (46), unlike Mms4 in budding yeast and human cells (42–44), clearly show differences in the mode of regulation of Mus81-Eme1/Mms4 among different organisms. They reflect distinct successful evolutionarily strategies and make important the study of these mechanisms in different systems.

Our results have indicated that the function of Mus81-Mms4 during the response to DNA damage is only executed after the bulk of genome replication is completed. Supporting this conclusion, we have additionally shown that new expression of Mus81-Mms4 significantly restores cell viability that is lost in the absence of this complex after MMS treatment, and that the function of the endonuclease is only carried out when the cells are at the end of S-phase, regardless of when the complex is newly synthesized. The post-replicative function of Mus81-Mms4 has a number of advantages that help to maintain genome stability. Thus, preventing its nucleolytic action during S-phase avoids the counterproductive cleavage of DNA structures during chromosome replication, like RFs. Furthermore, the low Mus81-Mms4 activity during S-phase, even when the DNA is damaged, avoids the undesired cleavage of DNA intermediates during DNA repair that can lead to chromosomal rearrangements and high levels of sister chromatid exchange. Indirectly, the absence of high Mus81-Mms4 activity in S-phase favours the action of the non-nucleolytic resolution pathway mediated by Sgs1-Top3-Rmi1, which does not originate crossovers or potential chromosome reorganizations. Likewise, timely activation of Mus81-Mms4 provides a safeguard mechanism that guarantees, just before chromosome segregation, the resolution of DNA intermediates that cannot be resolved by other proteins and need to be processed for proper completion of DNA repair and chromosome replication. This nucleolytic action can still put cells at some risk of genomic instability, but it constitutes the necessary last resort to resolve persistent intermediates that in any case, under non-pathological situations, should not be high in number. Finally, as the Mus81-Mms4 function is uncoupled from bulk genome replication, it is also expected that the proposed role of Mus81-Eme1/Mms4 in post-replication gap repair (11) is facilitated.

As the key aspects of the regulation of Mus81-Eme1/Mms4 in an unperturbed cell cycle seem to be conserved between budding yeast and human mitotic cells (42), it is likely that the main results presented in this work can be extrapolated to human cells suffering DNA damage. The correct function of Mus81-Eme1/Mms4 is necessary to prevent genomic instability, a hallmark of cancer (75). Although it is unclear whether Mus81 is important to avoid tumorigenesis (19,20), Mus81-associated defects have been linked to some types of cancers (76,77), and this endonuclease has been involved in oncogene-induced genotoxicity (78,79). Therefore, it would be interesting to study deeper whether the regulation of this endonuclease fails in some tumour cells. Future work will be necessary to explore the possible potential of Mus81-Eme1 as a tumour marker and to gain information that may help in the improvement of anti-cancer therapy.

## SUPPLEMENTARY DATA

Supplementary Data are available at NAR Online, including [43,47,48,61].

Supplementary Data
