# Supplementary material for: Temporal regulation of the Mus81-Mms4 endonuclease ensures cell survival under conditions of DNA damage
Source: Nucleic Acids Res. 2013 Jul 30;41(19):8943–58. doi: 10.1093/nar/gkt645 (PMC3799426; doi:10.1093/nar/gkt645)
Supplement: Supplementary Data [file supp_41_19_8943__index.html]

Temporal regulation of the Mus81-Mms4 endonuclease ensures cell survival under conditions of DNA damage — Temporal regulation of the Mus81-Mms4 endonuclease ensures cell survival under conditions of DNA damage — Supplementary Data 

# Temporal regulation of the Mus81-Mms4 endonuclease ensures cell survival under conditions of DNA damage

## 

files

**Files in this Data Supplement:**

- Supplementary Data - pdf file
