# Supplementary material for: Temporal regulation of the Mus81-Mms4 endonuclease ensures cell survival under conditions of DNA damage
Source: Nucleic Acids Res. 2013 Jul 30;41(19):8943–58. doi: 10.1093/nar/gkt645 (PMC3799426; doi:10.1093/nar/gkt645)
Supplement: Supplementary Data [file supp_gkt645_nar-01380-d-2013-File008.pdf]

Supplementary Table S1. Saugar et al.

Strain	Relevant genotype	Source
YMV17	*MAT***a** *mus81Δ::kanMX4 ARS608Δ::HIS3 ARS609Δ::TRP1 ade2-1::ADE2*	This work
YMV22	*MAT***a** *mus81Δ::kanMX4 TUB1-GFP::LEU2 ARS608Δ::HIS3 ARS609Δ::TRP1 ade2-1::ADE2*	This work
YMV33	*MAT***a** P_{ADH1}*-3HA-MMS4::natNT2*	(43)
YMV39	*MAT***a** *mus81Δ::kanMX4*	This work
YMV48	*MAT***a** *mms4Δ::URA3*	This work
YMV49	*MAT***a** *mus81Δ::kanMX4 mms4Δ::URA3*	This work
YJT110	*MAT***a** *ARS608Δ::HIS3 ARS609Δ::TRP1 ade2-1::ADE2*	(61)
YJT126	*MAT***a** *sml1Δ::URA3 TUB1-GFP::TRP1*	(61)
YJT127	*MAT***a** *rad53Δ::LEU2 sml1Δ::URA3 TUB1-GFP::TRP1*	(61)
YSG23	*MAT***a** *mus81::*$P_{GAL1,10}$*-3HA-MUS81::His3MX6 mms4::*$P_{GAL1,10}$*-3HA-MMS4::kanMX6*	This work
YSG24	*MAT***a** *mus81::*P_{ADH1}*-3HA-MUS81::natNT2 mms4::*P_{ADH1}*-3HA-MMS4::natNT2*	This work
YSG56	*MAT***a** *sgs1Δ::kanMX4 yen1Δ::LEU2 mms4::*P_{ADH1}*-3HA-MMS4::natNT2*	This work
YMG21	*MAT***a** *sgs1Δ::kanMX4 mms4::*P_{ADH1}*-3HA-MMS4::natNT2*	This work

Supplementary Table S1. *S. cerevisiae* strains used in this study. All the strains were constructed by standard techniques, using the pYM (47) or pML (48) plasmid series as templates for PCR. All the strains were based on W303-1a (*MAT***a** *ade2-1 ura3-1 his3-11,15 trp1-1 leu2-3,112 can1-100).*

References

43. Gallo-Fernández,M., Saugar,I., Ortiz-Bazán,M.A., Vázquez,M.V. and Tercero,J.A. (2012) Cell cycle-dependent regulation of the nuclease activity of Mus81-Eme1/Mms4. *Nucleic Acids Res.*, **40**, 8325-8335.

47. Janke,C., Magiera,M.M., Rathfelder,N., Taxis,C., Reber,S., Maekawa,H., Moreno-Borchart,A., Doenges,G., Schwob,E., Schiebel,E. *et al.* (2004) A versatile toolbox for PCR-based tagging of yeast genes: new fluorescent proteins, more markers and promoter substitution cassettes. *Yeast*, **21**, 947-962.

48. Longtine,M.S., McKenzie,A., 3rd, Demarini,D.J., Shah,N.G., Wach,A., Brachat,A., Philippsen,P. and Pringle,J.R. (1998) Additional modules for versatile and economical PCR-based gene deletion and modification in *Saccharomyces cerevisiae*. *Yeast*, **14**, 953-961.

61. Vázquez,M.V., Rojas,V. and Tercero,J.A. (2008) Multiple pathways cooperate to facilitate DNA replication fork progression through alkylated DNA. *DNA Repair (Amst)*, **7**, 1693-1704.

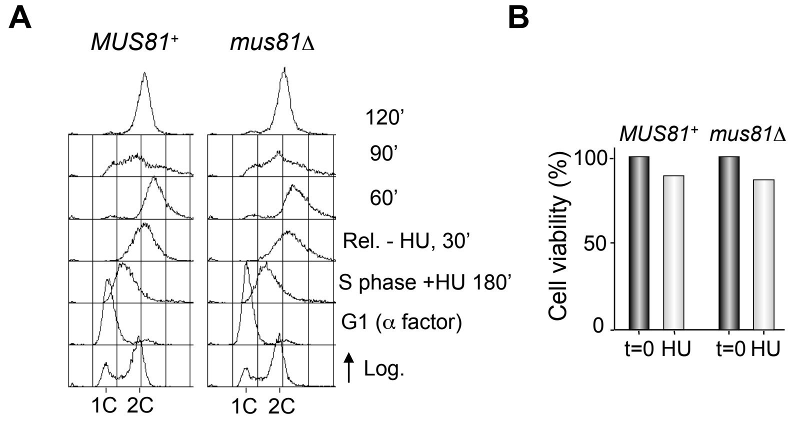

Supplementary Figure S1. Mus81-Mms4 is not required for coping with replicative stress induced by hydroxyurea (HU). (**A**) *MUS81*$^{+}$ control and *mus81*Δ cells were synchronized in G1 with α factor and then released for 180 min into fresh medium with 0.2 M HU. HU was then removed and the cells were allowed to progress through the cell cycle. Samples were taken at the indicated time points and the DNA content was analysed by flow cytometry to follow cell cycle progression. (**B**) Cell viability after the HU treatment.

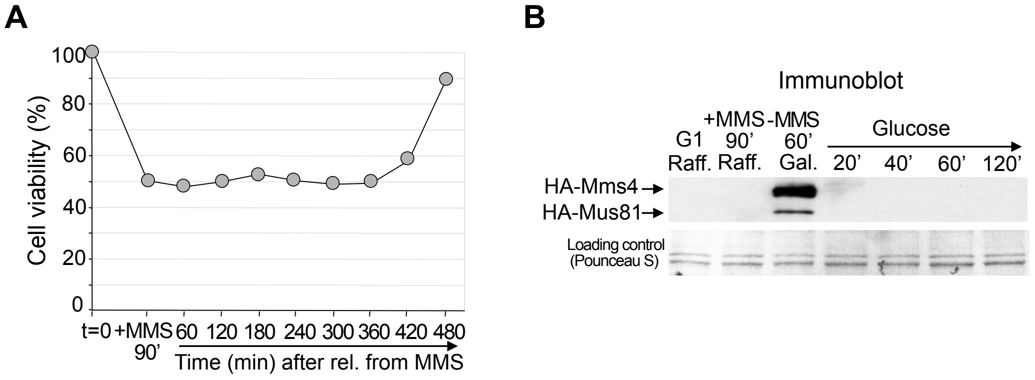

Supplementary Figure S2

(**A**) Cell viability is recovered immediately after MMS treatment when $P_{GAL1\text{-}10}$-*MUS81* $P_{GAL1\text{-}10}$-*MMS4* cells are plated on YP solid medium containing galactose. The cells were grown in medium with raffinose, synchronized in G1 with α factor and then released into medium containing raffinose plus 0.033% MMS. After 90 min, the MMS was removed and the culture was transferred to medium containing galactose. Cells were taken at the indicated time points and plated on YPGal, and cell viability was monitored throughout the experiment.

(**B**) Mus81-Mms4 expressed under the control of the *GAL1-10* promoter is degraded rapidly upon transferring cells to medium with glucose. $P_{GAL1\text{-}10}$-*MUS81* $P_{GAL1\text{-}10}$-*MMS4* cells were grown in medium with raffinose (*GAL1-10* promoter inactive), synchronized in G1 using α factor and then released into medium containing raffinose plus 0.033% MMS. After 90 min, the MMS was removed and the culture was transferred to medium with galactose (*GAL1-10* promoter ON) for 60 min, which allowed the expression of the Mus81 and Mms4 subunits (see immunoblot). The cells were then transferred to medium containing glucose (*GAL1-10* promoter OFF) and both Mus81 and Mms4 were immediately degraded.

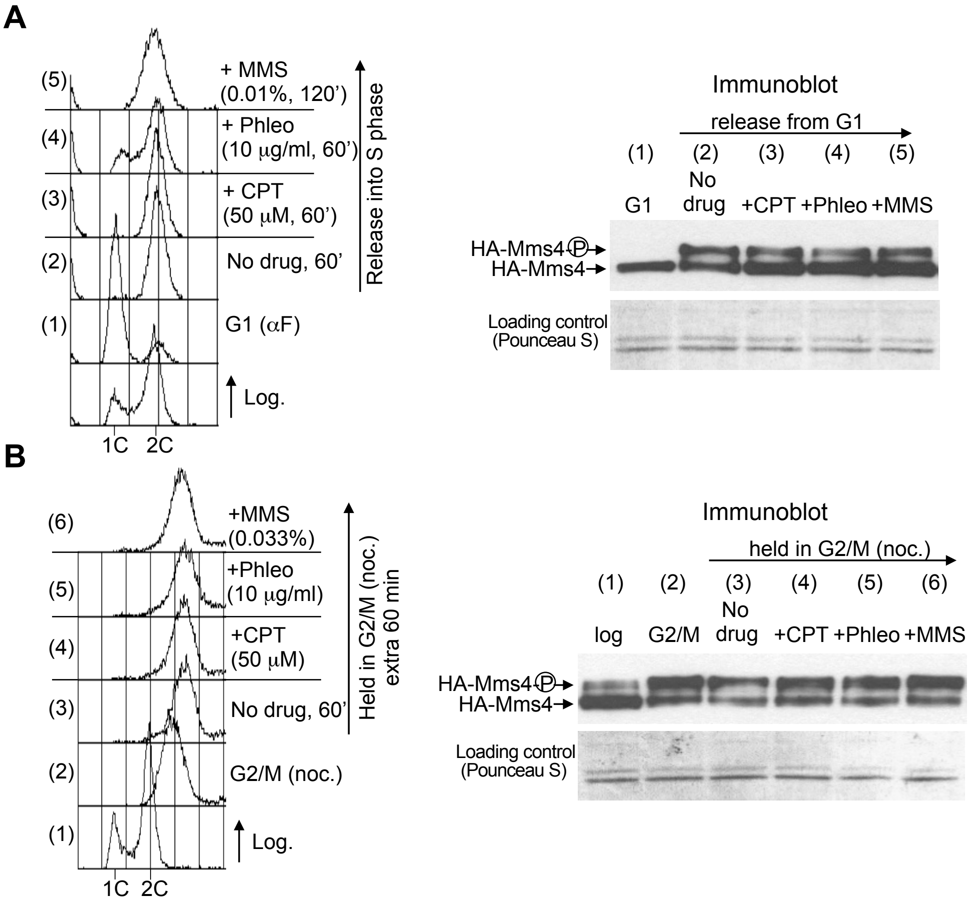

Supplementary Figure S3. Mms4 phosphorylation is not modified by the treatment of budding yeast cells with DNA-damaging agents.

(**A**) Mms4 phosphorylation is not modified when the cells are exposed to exogenous DNA damage during S-phase. *HA-MMS4* cells were synchronized in G1 phase with α factor (1). The culture was then divided into four parts, and in all cases the cells were released into S phase. One part was released in the absence of exogenous DNA damage, for 60 min (2). The other three were released in the presence of DNA-damaging drugs: 50 μM camptothecin (CPT), for 60 min (3); 10 μg/ml phleomycin (phleo), for 60 min (4) or 0.01% MMS, for 120 min (5). The DNA content was analysed by flow cytometry to follow cell cycle progression (left panel) and the phosphorylation of Mms4 was analysed by immunoblot (right panel). The data indicate that Mms4 hyperphosphorylation occurs when the cells reach a 2C DNA content (compare 1 with 2, 3, 4 and 5). The immunoblot also shows that Mms4 phosphorylation has the same pattern regardless of the presence or the absence of exogenous DNA damage (compare 2 with 3, 4 and 5).

(**B**) Mms4 phosphorylation is not modified when the cells are exposed to exogenous DNA damage in G2/M. Cycling *HA-MMS4* cells (1) were synchronized in G2/M with nocodazole (2). The culture was then divided into four parts, and in all cases the cells were held in G2/M for another 60 min. One part was not treated with any DNA-damaging agent (3). The other three were treated, respectively, with 50 μM camptothecin (CPT) (4); 10 μg/ml phleomycin (phleo) (5) or 0.033% MMS (6). The DNA content was analysed by flow cytometry to follow cell cycle progression (left panel) and the phosphorylation of Mms4 was analysed by immunoblot (right panel). The immunoblot shows that Mms4 phosphorylation does not undergo apparent changes when the cells are treated with any of the drugs tested (compare 2 and 3 with 4, 5 and 6).
